# Comparative Effectiveness of Sodium-Glucose Cotransporter 2 (SGLT-2) Inhibitors and Glucagon-like Peptide-1 (GLP-1) Receptor Agonists in Reducing Adverse Cardiovascular Events in Type 2 Diabetes

**DOI:** 10.7759/cureus.92048

**Published:** 2025-09-11

**Authors:** Syed Zakir Shah, Rahema Khan, Sadaf Moeez, Sanjay Kumar, Muhammad Umar Ejaz, Usman Aslam, Mian Waqar Mustafa, Mahwish Ashraf, Imtiaz Mustafa, Fahad Asim

**Affiliations:** 1 Endocrinology, Shifa International Hospitals Limited, Islamabad, PAK; 2 Medicine, Rasheed Hospital, Lahore, PAK; 3 Biological Sciences, International Islamic University, Islamabad, PAK; 4 Internal Medicine, Dr. Ruth K. M. Pfau Civil Hospital Karachi, Dow University of Health Sciences, Karachi, PAK; 5 Cardiology, National Institute of Cardiovasculer Diseases, Karachi, PAK; 6 College of Medicine, CMH (Combined Military Hospital) Lahore Medical College and Institute of Dentistry, Lahore, PAK; 7 Pharmacology, CMH (Combined Military Hospital) Lahore Medical College and Institute of Dentistry, Lahore, PAK; 8 Pharmacy, Forman Christian College University, Lahore, PAK; 9 Diabetes and Endocrinology, Wah Medical College, Wah Cantonment, PAK; 10 Institute of Molecular Biology and Biotechnology/Centre for Research In Molecular Medicine, The University of Lahore, Lahore, PAK; 11 Pharmacology and Therapeutics/Clinical Pharmacy and Pharmacy Practice, Faculty of Pharmacy, The University of Lahore, Lahore, PAK

**Keywords:** cardiovascular outcomes, glp-1 receptor agonists, sglt-2 inhibitors, type 2 diabetes, weight loss

## Abstract

Background

Cardiovascular outcome trials have demonstrated that sodium-glucose cotransporter-2 inhibitors (SGLT-2i) and glucagon-like peptide-1 receptor agonists (GLP-1 RA) reduce cardiovascular risk in patients with type 2 diabetes (T2D). However, their comparative effectiveness remains uncertain, particularly in South Asian populations.

Objective

The objective of this study was to compare the effectiveness of SGLT-2i and GLP-1 RA in reducing major adverse cardiovascular events (MACE) and improving cardiometabolic parameters in patients with T2D.

Methods

This retrospective study was conducted at the Combined Military Hospital, Lahore, Pakistan, over 24 months. A total of 200 patients with T2D were included, with 100 in the SGLT-2i group and 100 in the GLP-1 RA group. Baseline characteristics, including age, sex, duration of diabetes, HbA1c, body mass index (BMI), hypertension, and dyslipidemia, were comparable between groups. The primary outcome was the incidence of MACE, while secondary outcomes included changes in HbA1c, blood pressure, lipid profile, and BMI. Statistical analysis included chi-square tests, independent t-tests, Kaplan-Meier survival curves, and Cox regression.

Results

Over a mean follow-up of 18.6 ± 3.1 months, MACE occurred in 18% of patients in the SGLT-2i group compared to 30% in the GLP-1 RA group (χ² = 4.26, p = 0.04). Kaplan-Meier analysis showed higher event-free survival with SGLT-2i (log-rank χ² = 4.71, p = 0.03). Cox regression revealed a 42% reduced risk of MACE with SGLT-2i (HR 0.58, 95% CI 0.35-0.95, p = 0.03). Secondary outcomes indicated greater reductions in systolic blood pressure (p = 0.02) and BMI (p = 0.01) with SGLT-2i, while HbA1c reduction was slightly greater with GLP-1 RA but not statistically significant (p = 0.42). Lipid changes favored GLP-1 RA but were not significant.

Conclusion

SGLT-2 inhibitors demonstrated superior effectiveness compared to GLP-1 receptor agonists in reducing cardiovascular risk, lowering systolic blood pressure, and promoting weight loss in Pakistani patients with T2D. These findings support the preferential use of SGLT-2i in high cardiovascular risk populations.

## Introduction

Cardiovascular outcome trials (CVOTs) conducted over the past few years in patients with type 2 diabetes (T2D) have demonstrated that both glucagon-like peptide-1 receptor agonists (GLP-1 RA) and sodium-glucose cotransporter-2 inhibitors (SGLT-2i) significantly reduce the risk of major adverse cardiovascular events (MACE), all-cause mortality, and progression of nephropathy [1,2]. Despite these shared benefits, the two drug classes differ in their specific cardiovascular and safety profiles. In placebo-controlled trials, GLP-1 RA were associated with a notable reduction in stroke risk, while SGLT-2i had a neutral effect on stroke but conferred greater protection against hospitalization for heart failure and adverse renal outcomes [3,4]. With respect to safety and tolerability, gastrointestinal side effects are more frequent with GLP-1 RA [5], whereas SGLT-2i are associated with a higher incidence of genital infections, volume depletion, and ketoacidosis, occasionally necessitating treatment discontinuation [6].

Evidence from previous studies has largely confirmed the efficacy and safety findings of these two drugs, observed in CVOTs [1,2]. Current international guidelines recommend initiating therapy with either GLP-1 RA or SGLT-2i in patients with established atherosclerotic cardiovascular disease or high cardiovascular risk, regardless of baseline glycemic control [7]. Among these, SGLT-2i are particularly emphasized in patients with coexisting heart failure, chronic kidney disease, or albuminuria [8]. However, no head-to-head randomized controlled trials are currently available to directly compare the cardiovascular outcomes of GLP-1 RA and SGLT-2i. In this context, previous research data, though limited by lack of randomization, potential bias, and data heterogeneity [9], remain essential to evaluate their comparative effectiveness. Indeed, several studies conducted across different populations have suggested comparable overall benefits of both agents on composite cardiovascular endpoints [10-12].

Given these considerations, there is a pressing need to generate context-specific evidence on the comparative effectiveness of SGLT-2i and GLP-1 RA, particularly in South Asian populations, where cardiovascular risk profiles and treatment responses may differ from Western cohorts. The rationale of this study is to provide real-world comparative evidence on the cardiovascular effectiveness of these two drug classes in Pakistani patients with T2D. Therefore, the objective of this study is to evaluate and compare the effectiveness of SGLT-2 inhibitors and GLP-1 receptor agonists in reducing adverse cardiovascular events among patients with T2D treated at a tertiary care center in Lahore, Pakistan.

## Materials and methods

This retrospective, observational cohort study was conducted at the Department of Medicine, Combined Military Hospital (CMH), Lahore, Pakistan, over a 24-month period from January 2022 to December 2023. Data were retrieved from the hospital’s electronic medical records and pharmacy dispensing system. Patients were divided into two groups: Group A included patients prescribed SGLT-2 inhibitors, while Group B included those prescribed GLP-1 receptor agonists. Since the study was retrospective and based entirely on previously collected medical records, the requirement for informed consent was waived, and ethical approval was exempted by the institutional review process. Patient confidentiality was maintained through anonymized coding.

Eligibility criteria

Purposive sampling was employed to identify eligible patients. Inclusion criteria were: patients aged 40-75 years, with a confirmed diagnosis of T2D for at least five years, on either SGLT-2 inhibitors or GLP-1 receptor agonists for at least 12 months, and with complete follow-up records available for one year after initiation of therapy. Exclusion criteria were: patients with type 1 diabetes, stage 4 or higher chronic kidney disease at baseline, active malignancy, simultaneous use of both drug classes, non-adherence documented in medical records, or incomplete follow-up data.

Sample size calculation

Sample size was calculated using the WHO sample size calculator, with a 95%CI, power of 80%, and an expected 15% difference in cardiovascular event rates between the two groups as per prior literature. This yielded a minimum of 180 patients (90 in each group). To allow for incomplete data, 200 patients were included, with 100 in each group.

Outcomes

The primary outcome was the incidence of MACE, defined as a composite of nonfatal myocardial infarction, nonfatal stroke, hospitalization for heart failure, and cardiovascular death. Secondary outcomes included change in HbA1c, systolic and diastolic blood pressure, lipid profile (total cholesterol, low-density lipoprotein-cholesterol (LDL-C), high-density lipoprotein-cholesterol (HDL-C), triglycerides), and body mass index (BMI) from baseline to 12 months.

Data collection

HbA1c was measured using high-performance liquid chromatography (HPLC), lipid profile was assessed enzymatically using an automated analyzer, and blood pressure was recorded by a calibrated sphygmomanometer (mean of two seated readings per visit). BMI was calculated as weight in kilograms divided by height in meters squared. Medication adherence was verified through prescription refill logs and physician notes.

Data analysis

All statistical analyses were performed using IBM SPSS Statistics for Windows, version 26 (IBM Corp., Armonk, New York, United States). Continuous variables, including age, duration of diabetes, HbA1c, BMI, systolic and diastolic blood pressure, and lipid parameters, were expressed as mean ± standard deviation (SD) and compared between the two groups using independent sample t-tests. Categorical variables, such as gender, smoking status, presence of hypertension, dyslipidemia, and occurrence of MACE, were presented as frequencies and percentages and compared using chi-square tests. Time-to-event outcomes for MACE were analyzed using Kaplan-Meier survival analysis, and differences between the two groups were assessed using the log-rank test. Cox proportional hazards regression analysis was applied to adjust for potential confounders, including age, sex, baseline HbA1c, duration of diabetes, hypertension, and dyslipidemia. A p-value of <0.05 was considered statistically significant.

## Results

A total of 200 patients with T2D were included, with 100 in the SGLT-2 inhibitor group and 100 in the GLP-1 receptor agonist group. The mean follow-up duration was 18.6 ± 3.1 months, with no significant difference between groups (t = 0.82, p = 0.41). The baseline demographic and clinical characteristics were comparable between the two groups (Table 1). No statistically significant differences were observed in age, sex distribution, duration of diabetes, baseline HbA1c, BMI, hypertension, dyslipidemia, or smoking status.

**Table 1:** Baseline demographic and clinical characteristics of study participants Data are presented as n (%) except for age, which is presented as mean ± SD. Independent sample t-test used for continuous variables; chi-square test used for categorical variables. p < 0.05 is considered statistically significant. ASCVD: atherosclerotic cardiovascular disease; CKD: chronic kidney disease; eGFR: estimated glomerular filtration rate; ACEi: angiotensin-converting enzyme inhibitor; ARB: angiotensin receptor blocker; SGLT-2i: sodium-glucose cotransporter-2 inhibitor; GLP-1 RA: glucagon-like peptide-1 receptor agonist

Variable	SGLT-2i Group (n=100)	GLP-1 RA Group (n=100)	Test Statistic	p-value
Age (years), mean ± SD	59.7 ± 8.1	58.9 ± 8.3	t = 0.61	0.54
Male sex, n (%)	56 (56%)	52 (52%)	χ² = 0.26	0.61
Female sex, n (%)	44 (44%)	48 (48%)	χ² = 0.26	0.61
Duration of diabetes (years), mean ± SD	9.8 ± 3.4	10.1 ± 3.2	t = -0.73	0.47
Baseline HbA1c (%), mean ± SD	8.5 ± 1.1	8.6 ± 1.0	t = -0.49	0.62
BMI (kg/m²), mean ± SD	28.7 ± 3.5	29.0 ± 3.6	t = -0.56	0.58
Hypertension, n (%)	64 (64%)	67 (67%)	χ² = 0.17	0.68
Dyslipidemia, n (%)	59 (59%)	62 (62%)	χ² = 0.15	0.70
Current smokers, n (%)	22 (22%)	19 (19%)	χ² = 0.28	0.59
ASCVD, n (%)	31 (31%)	28 (28%)	χ² = 0.19	0.66
Heart failure, n (%)	18 (18%)	16 (16%)	χ² = 0.13	0.72
CKD (eGFR <60 mL/min/1.73m²), n (%)	14 (14%)	17 (17%)	χ² = 0.37	0.54
Albuminuria, n (%)	21 (21%)	19 (19%)	χ² = 0.10	0.75
Insulin use, n (%)	35 (35%)	38 (38%)	χ² = 0.19	0.66
Statin use, n (%)	71 (71%)	74 (74%)	χ² = 0.19	0.66
ACEi/ARB use, n (%)	62 (62%)	65 (65%)	χ² = 0.18	0.67

Primary outcome: MACE

Over the follow-up period, MACE occurred in 18% of patients in the SGLT-2i group and 30% in the GLP-1 RA group (χ² = 4.26, p = 0.04). Kaplan-Meier survival analysis demonstrated significantly higher event-free survival in the SGLT-2 inhibitor group (log-rank χ² = 4.71, p = 0.03). Cox regression analysis, adjusted for confounders, showed that SGLT-2i use reduced the hazard of MACE by 42% (hazard ratio (HR): 0.58, 95% CI: 0.35-0.95, p = 0.03) (Table 2).

**Table 2:** Comparison of major adverse cardiovascular events between groups p < 0.05 is considered statistically significant. SGLT-2i: sodium-glucose cotransporter-2 inhibitor; GLP-1 RA: glucagon-like peptide-1 receptor agonist

Outcome	SGLT-2i Group (n=100), n (%)	GLP-1 RA Group (n=100), n (%)	Test Statistic	p-value
Any MACE, n (%)	18 (18%)	30 (30%)	χ² = 4.26	0.04
Nonfatal myocardial infarction	6 (6%)	10 (10%)	χ² = 1.12	0.29
Nonfatal stroke	5 (5%)	8 (8%)	χ² = 0.75	0.39
Hospitalization for heart failure	4 (4%)	9 (9%)	χ² = 1.95	0.16
Cardiovascular death	3 (3%)	7 (7%)	χ² = 1.62	0.20

Secondary outcomes

Both groups showed significant improvements in glycemic and metabolic parameters. HbA1c reduction was slightly greater in the GLP-1 group, though not statistically significant (t = 0.81, p = 0.42). SGLT-2 inhibitors led to greater reductions in systolic BP (t = -2.35, p = 0.02) and BMI (t = -2.65, p = 0.01). Lipid profile changes were more favorable in the GLP-1 group, though not statistically significant (Table 3).

**Table 3:** Changes in glycemic and cardiometabolic parameters from baseline to 12 months Data are presented as mean ± SD. Independent sample t-test was used. p < 0.05 is considered statistically significant. SGLT-2i: sodium-glucose cotransporter-2 inhibitor; GLP-1 RA: glucagon-like peptide-1 receptor agonist

Parameter	SGLT-2i Group (n=100), Baseline → 12 Months	GLP-1 RA Group (n=100), Baseline → 12 Months	Test Statistic	p-value
HbA1c (%)	8.5 ± 1.1 → 7.4 ± 0.9	8.6 ± 1.0 → 7.3 ± 0.8	0.81	0.42
Systolic BP (mmHg)	138.6 ± 12.3 → 128.2 ± 10.7	137.8 ± 11.9 → 132.6 ± 11.2	-2.35	0.02
Diastolic BP (mmHg)	85.2 ± 7.4 → 80.1 ± 6.9	85.6 ± 7.1 → 82.0 ± 7.3	-1.71	0.09
Total cholesterol (mg/dL)	211.4 ± 35.2 → 195.3 ± 31.8	213.1 ± 33.7 → 189.7 ± 29.5	1.25	0.21
LDL-C (mg/dL)	129.5 ± 28.1 → 118.7 ± 24.5	128.6 ± 27.3 → 114.1 ± 22.9	1.11	0.27
HDL-C (mg/dL)	42.1 ± 6.2 → 45.7 ± 6.8	41.8 ± 6.4 → 46.3 ± 7.1	-0.69	0.49
Triglycerides (mg/dL)	183.2 ± 42.7 → 168.5 ± 38.4	185.6 ± 43.2 → 166.4 ± 37.9	0.48	0.63
BMI (kg/m²)	28.7 ± 3.5 → 27.1 ± 3.2	29.0 ± 3.6 → 28.4 ± 3.4	-2.65	0.01

Survival analysis and Cox regression

Kaplan-Meier survival curves showed higher MACE-free survival in the SGLT-2 inhibitor group (log-rank χ² = 4.71, p = 0.03). Cox regression confirmed SGLT-2 inhibitor use as an independent protective factor against MACE (HR = 0.58, 95% CI: 0.35-0.95, p = 0.03) after adjustment for confounders (Table 4).

**Table 4:** Cox proportional hazards regression analysis for predictors of MACE Data presented as HR with 95% CI. Cox proportional hazards regression was used. p < 0.05 is considered statistically significant. MACE: major adverse cardiovascular events

Variable	Hazard Ratio (HR)	95% CI	Wald χ²	p-value
SGLT-2 inhibitor use	0.58	0.35 – 0.95	4.65	0.03
Age (per year increase)	1.02	0.99 – 1.05	2.54	0.11
Male sex	1.08	0.66 – 1.78	0.11	0.74
Duration of diabetes	1.04	0.98 – 1.09	1.85	0.17
Baseline HbA1c	1.12	0.93 – 1.35	1.58	0.21
Hypertension	1.39	0.82 – 2.34	1.57	0.21
Dyslipidemia	1.28	0.75 – 2.20	0.87	0.35

## Discussion

In this retrospective comparative analysis, we evaluated the effectiveness of SGLT-2i versus GLP-1 RA in reducing adverse cardiovascular events among patients with T2D in a tertiary care setting in Pakistan. Our findings demonstrated that while both agents improved cardiometabolic risk factors, SGLT-2i were associated with a significantly lower incidence of MACE and greater reductions in systolic blood pressure and BMI compared to GLP-1 receptor agonists.

The baseline demographic and clinical characteristics of our study population were well-balanced between treatment groups, minimizing confounding by indication. Both groups had comparable mean age, sex distribution, duration of diabetes, HbA1c, BMI, and prevalence of comorbid hypertension and dyslipidemia. This is consistent with prior real-world studies that matched GLP-1 RA and SGLT-2i cohorts to allow robust outcome comparisons [13,14]. The comparability of baseline characteristics strengthens the validity of the differences observed in outcomes.

The primary outcome analysis revealed that SGLT-2i reduced the incidence of MACE (18%) compared with GLP-1 RA (30%), with survival analysis further confirming superior event-free survival in the SGLT-2i group. Cox regression indicated a 42% risk reduction in MACE with SGLT-2i after adjusting for confounders. These results align with evidence from large-scale CVOTs, such as Empagliflozin Cardiovascular Outcome Event Trial in Type 2 Diabetes Mellitus Patients - Removing Excess Glucose (EMPA-REG OUTCOME) [15] and Canagliflozin Cardiovascular Assessment Study (CANVAS) [16], which consistently demonstrated robust cardioprotective effects of SGLT-2 inhibitors, particularly in reducing hospitalization for heart failure and cardiovascular mortality.** **In contrast, GLP-1 RA has shown more consistent benefits in reducing atherosclerotic outcomes such as stroke, as seen in published literature [2,3]. However, our study did not detect a significant difference in nonfatal stroke or myocardial infarction between the two groups, likely due to the relatively smaller sample size and shorter follow-up duration compared to CVOTs.

Similar findings have been reported in other real-world analyses, including Patorno et al. [12] and Sattar et al. [17], which suggested that both drug classes exert comparable protective effects on broad composite cardiovascular endpoints, though class-specific strengths may differ. It is also important to note that EMPA-REG OUTCOME [15] and Liraglutide Effect and Action in Diabetes: Evaluation of Cardiovascular Outcome Results (LEADER) [18] trials were placebo-controlled CVOTs rather than head-to-head comparisons. Therefore, differences in absolute risk reduction observed across these trials cannot be directly compared, as variations in study design, baseline population risk, and follow-up duration may influence outcomes. While both classes demonstrated significant reductions in major adverse cardiovascular events, conclusions about comparative efficacy require cautious interpretation and ideally should be drawn from dedicated head-to-head studies.

With respect to secondary outcomes, both groups achieved clinically meaningful reductions in HbA1c, consistent with their known glucose-lowering efficacy. The slightly greater HbA1c reduction observed in the GLP-1 RA group did not reach statistical significance, paralleling findings from head-to-head glycemic efficacy studies, where GLP-1 RA generally showed superior glucose-lowering capacity [19,20]. Our observation that SGLT-2 inhibitors induced greater reductions in systolic blood pressure is well supported by prior research; SGLT-2i promote osmotic diuresis, natriuresis, and vascular effects that lead to sustained blood pressure lowering [21,22]. Similarly, the more pronounced reduction in BMI with SGLT-2i in our cohort reflects its established mechanism of inducing modest weight loss through glycosuria and negative energy balance [23]. While GLP-1 RA are also associated with weight loss due to appetite suppression and delayed gastric emptying, differences between the two groups in our study may reflect heterogeneity in adherence, drug selection within class, or dietary behaviors.

Changes in lipid parameters were directionally favorable in both groups but not statistically significant between them. GLP-1 RA users showed a slightly greater reduction in total cholesterol and LDL-C in our study, consistent with a review by Premji et al. demonstrating modest lipid benefits with GLP-1 RA compared to SGLT-2i [24]. Improvements in HDL-C and triglycerides were comparable, in line with prior real-world observations [25]. Given the relatively small sample size, our study may have been underpowered to detect subtle intergroup differences in lipid outcomes.

The superior MACE-free survival observed with SGLT-2i in our study is congruent with the accumulating evidence from both randomized and real-world studies. Data from CVD-REAL (Comparative Effectiveness of Cardiovascular Outcomes in New Users of Sodium-Glucose Cotransporter-2 Inhibitors) [26] and EASEL (Evidence for Cardiovascular Outcomes With Sodium Glucose Cotransporter 2 Inhibitors in the Real World) [27] real-world cohorts have shown lower risks of hospitalization for heart failure and all-cause mortality with SGLT-2i compared to other glucose-lowering drugs. Meanwhile, GLP-1 RA have consistently reduced stroke incidence in trials such as REWIND (Researching cardiovascular Events with a Weekly Incretin in Diabetes) [28], though this was not observed in our population. This discrepancy may relate to ethnic and regional differences in cardiovascular risk phenotypes; South Asian populations, including Pakistan, are known to have a higher prevalence of heart failure and cardiometabolic clustering, which may favor SGLT-2i over GLP-1 RA in real-world practice [29].

There are limitations to this study that merit consideration. As a retrospective single-center study, it is subject to residual confounding and potential selection bias. The modest sample size and 24-month follow-up may have limited the detection of less frequent cardiovascular endpoints such as stroke and cardiovascular death. Additionally, drug-specific subgroup analyses (e.g., empagliflozin vs dapagliflozin; liraglutide vs semaglutide) were not performed due to sample size constraints, which may have masked intraclass differences.

## Conclusions

Our study provides real-world evidence that SGLT-2 inhibitors may confer superior cardiovascular protection compared to GLP-1 receptor agonists in patients with type 2 diabetes, particularly with respect to reducing major adverse cardiovascular events, lowering blood pressure, and promoting weight loss. These findings complement prior CVOTs and real-world studies, reinforcing guideline recommendations to prioritize SGLT-2i in patients with T2D and high cardiovascular or renal risk. Future prospective multicenter studies with larger sample sizes and longer follow-up are warranted to confirm these comparative benefits and explore ethnic-specific responses to therapy.
